# Absence of Anus

**Published:** 1898-02

**Authors:** D. L. Peeples

**Affiliations:** Navasota, Texas


### ABSENCE OF ANUS.

Lucinda, (colored) age 23, wife of Ed Kemper, age 37, residing three miles east of Navasota, Texas, and mother of five children. First child had six fingers on left hand, lived four years and six months. Second child had twenty-four fingers and toes, lived only one day. Third child well formed in every respect and still living. Fourth child had twenty-two fingers and toes, died in infancy. Husband's sister was delivered of a child with twenty-four fingers and toes, died at the age of four years. Fifth child born January 10th, 1898 (full term), with complete absence of Anus and no indication of rectum, partially deformed penis, large and well formed scrotum. Had four fingers on right hand, three being well proportioned, but index abnormally large, with poor articulations, and protruding at right angles anteriorly from the hand. Index on left hand large and with peculiar morphology somewhat similar to right, also half thumb above index on same hand. Right foot had only four toes, but well formed; left foot had three fairly well formed toes and two little toes well proportioned, one exactly above the other. The child sustained further deformity in both feet, congenital talipes calcaneo-valgus, was also badly hair-lipped and had protruding eyes. Considerable redundancy of tissue was to be found in the dextral upper lip with prominence of both maxillaries just at this point, and breaking suddenly into deep congenital fissure, extending slightly into the left nasal cavity. This condition rendered it unable to nurse; however, it took nourishment up to the eighth day, when I first saw it. The kidneys acted all along. I would have attempted an operation for artificial anus, but realizing the approaching end, desisted. On account of inclemency of the weather, I was unable to photograph the child. Therefore I had my friend, Dr. E. A. Harris of this place to accompany me that we might persuade the parents to give us possession of the child on the day of its expiration, which occurred on the 19th day of January, 1898, nine days after birth. However, our entreaties were unsuccessful.

D. L. PEEPLES. M. D.

Navasota, Texas, January 20, 1898.

